# Zr-Based Metal-Organic Frameworks for Green Biodiesel Synthesis: A Minireview

**DOI:** 10.3390/bioengineering9110700

**Published:** 2022-11-17

**Authors:** Qiuyun Zhang, Jialu Wang, Shuya Zhang, Juan Ma, Jingsong Cheng, Yutao Zhang

**Affiliations:** 1 College Rural Revitalization Research Center of Guizhou, Anshun University, Anshun 561000, China; 2 School of Chemistry and Chemical Engineering, Anshun University, Anshun 561000, China; 3 School of Resource and Environmental Engineering, Anshun University, Anshun 561000, China

**Keywords:** Zr-based MOFs, heterogeneous catalysis, esterification, transesterification, biofuels

## Abstract

Metal–organic frameworks (MOFs) have widespread application prospects in the field of catalysis owing to their functionally adjustable metal sites and adjustable structure. In this minireview, we summarize the current advancements in zirconium-based metal–organic framework (Zr-based MOF) catalysts (including single Zr-based MOFs, modified Zr-based MOFs, and Zr-based MOF derivatives) for green biofuel synthesis. Additionally, the yields, conversions, and reusability of Zr-based MOF catalysts for the production of biodiesel are compared. Finally, the challenges and future prospects regarding Zr-based MOFs and their derivatives for catalytic application in the biorefinery field are highlighted.

## 1. Introduction

With rapid population growth and high industrial energy demand from fossil fuel resources, fossil fuel usage leading to the energy security crisis and climate change (e.g., greenhouse gas emissions) has been of great concern [1]. Based on this, it is necessary to search for alternative fuel resources. Currently, renewable biofuels derived from biomass have gained enormous attention, and one of these liquid biofuels is biodiesel [2]. Chemically, biodiesel (fatty acid alkyl ester, FAME) is generally produced via the transesterification of edible oils (e.g., rapeseed oil, palm oil, sunflower oil), non-edible oils (e.g., *Jatropha curcus*, *Euphorbia lathyris* L., *Monotheca buxifolia*, *Sinapis Arvensis*), microalgal oils, or waste cooking oil or via the esterification of free fatty acids (e.g., oleic acid, lauric acid, palmitic acid) and methanol or ethanol using acid/alkali as catalysts [3,4,5,6,7,8].

**Figure 1:**
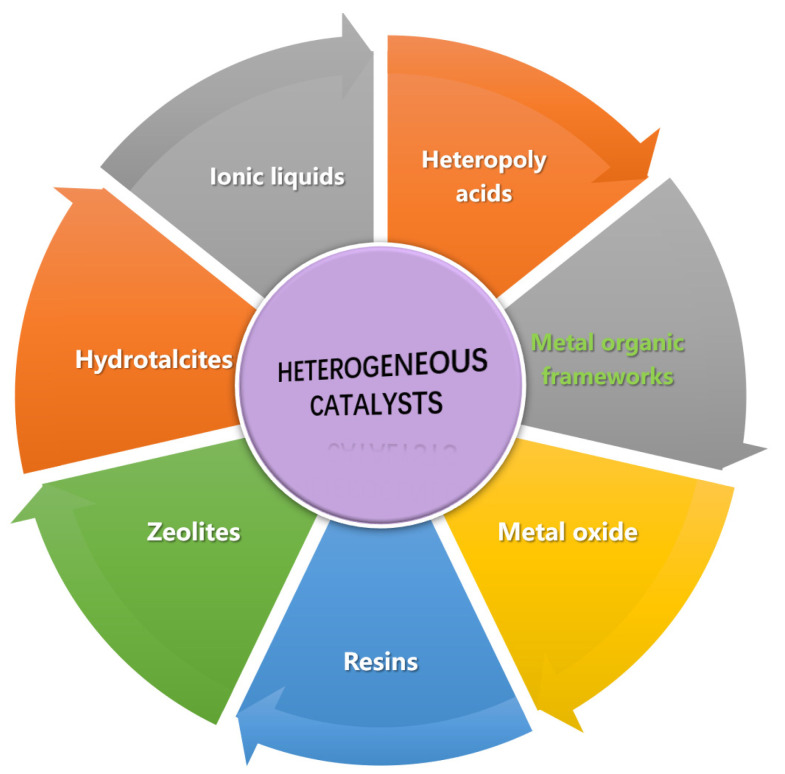
Various heterogeneous catalysts.

Traditionally, biodiesel production is carried out using liquid acid/alkali catalysts due to their high catalytic activity. Unfortunately, these homogeneous catalytic processes exhibit numerous disadvantages, such as high operating costs for steps such as product purification, catalyst neutralization, and a large amount of industrial wastewater that requires treatment [9]. With regard to this, the utilization of heterogeneous catalysts is becoming an efficient candidate for the production of biodiesel, because of their simple recovery, ease of reuse, insolubility in reaction solvents, and reduction in waste treatment [10]. According to Figure 1, various kinds of heterogeneous acid/alkali catalysts are available for different types of organic reactions, including metal oxides, ionic liquids, heteropoly acids, zeolites, sulfonic-acid-functionalized catalysts, etc. [11,12]. However, some problems such as leaching, lesser activity, low stability, and longer reaction time for some heterogeneous catalysts are found in the transesterification/esterification reaction process [13].

Quite recently, metal–organic frameworks (MOFs) have gained enormous attention due to their unique features, such as great specific surface area, uniformity in pore size, large porosity, adjustable properties, tunable structures, and controllable functional groups [14]. In addition, MOFs are suitable to be functionalized by coordinating acid/base functional groups, which have been widely studied in catalysis [15,16,17]. Among the numerous types of MOFs, zirconium-based MOFs (Zr-based MOFs) have been frequently applied as potential porous materials owing to the presence of Lewis and Brønsted acidity [18,19]. At present, several reviews have summarized the applications of Zr-MOFs in catalysis [20,21]. However, none has given a detailed study on the applications of Zr-MOFs and derivatives for green biodiesel synthesis. Thus, the present review focuses on the current development of Zr-based MOFs and derivatives for green biofuel production. More importantly, the catalytic performance and reusability of single Zr-based MOFs, modified Zr-based MOFs, and Zr-based MOF derivatives are systematically discussed. Finally, the conclusions and prospects are emphasized.

## 2. Zr-Based MOF Catalysts

**Figure 2:**
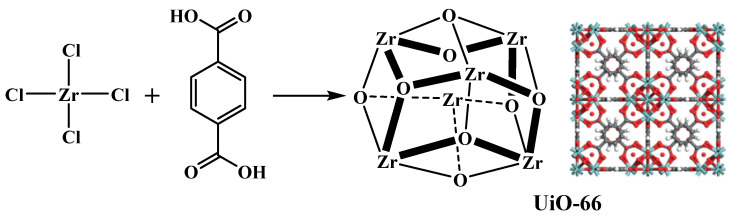
Schematic diagram of the structure and synthesis of UiO-66.

In 2008, a zirconium-based inorganic building brick (Zr-MOFs) was first reported by Lillerud et al. [22], and the results showed that Zr-MOFs possess high stability due to the combination of strong Zr-O bonds, the inner Zr_6_ cluster, and the addition of μ_3_-OH groups. A large number of Zr-based MOF catalysts have been widely used for fuel synthesis, including UiO-66 (see Figure 2), UiO-67, MOF-801, MOF-808, UiO-66-NH_2_, etc., owing to their excellent chemical and thermal stability under harsh conditions, large specific surface areas, smaller particle size, and strong acid sites by the tuning of structural defects [23,24].

### 2.1. Single Zr-Based MOF Catalysts

Single Zr-based MOFs usually show fewer Lewis acid characteristics due to the saturated Zr atom, and they have exhibited less catalytic activity in acid-catalyzed organic reactions. In view of this, the synthesis of various zirconium-containing UiO-66 samples by varying the synthesis temperatures and terephthalic acid/ZrCl_4_ ratios was reported by Zhou, et al. [25]. They found that UiO-66 catalysts with different amounts of defects could be synthesized under various synthesis conditions, and the catalytic activities of the UiO-66 depended on the defect amount. The obtained catalyst was used to catalyze the transesterification of soybean oil with methanol, and a conversion rate of 98.5% was acquired through a catalyst amount of 9%, with an oil/methanol molar ratio of 1:40, and at 140 °C for 5 h. UiO-66 with defects can be especially easily reused. Caratelli and his co-workers [26] also utilized UiO-66 MOFs as an acid catalyst for the production of ethyl levulinate via esterification from levulinic acid and ethanol. Various defective hydrated and dehydrated UiO-66 materials demonstrated excellent performance, and a maximum yield (>70%) of ethyl levulinate was achieved.

**Figure 3:**
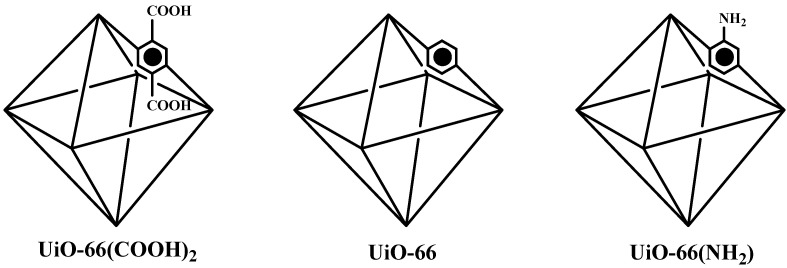
The suggested structures of UiO-66, UiO-66(COOH)_2_, and UiO-66(NH_2_).

A similar study was conducted by Jrad, et al. [27], in which three isostructural Zr-based MOFs (UiO-66, UiO-66(COOH)_2_, and UiO-66(NH_2_)) were prepared (Figure 3). UiO-66(COOH)_2_ demonstrated superior catalytic activity in the esterification of butyric acid and butanol, and a 90% conversion rate of butyl butyrate was achieved. This better catalytic activity was possibly related to the smaller particle size of the catalyst and the additional active acid functional groups grafted onto the original organic linker.

Wei’s group [28] also designed a series of defective UiO-66 catalysts for the esterification of levulinic acid with ethanol. The results showed that the synergistic effects between unsaturated Zr_6_ nodes and hydroxyl groups can have a significant influence on catalytic activity. The synthesized defective UiO-66 catalyst possessed excellent stability and could retain 75% of its initial activity after five cycles. Similarly, Chaemchuen and co-workers [29] synthesized UiO-66 catalyst for the esterification reaction between oleic acid and methanol. According to the kinetic analysis, an activation energy of 54.9 ± 1.8 kJ/mol was obtained, and the desorption of methyl oleate was found to be irreversible. Desidery, et al. [30] tested MOF-808 for the conversion of dimethyl carbonate into ethyl methyl carbonate. The MOF-808 catalyst exhibited superior catalytic performance and could be recycled for up to four cycles without any major change in activity or structure. Shaik, et al. [31] developed the Zr-fumarate MOF (MOF-801) as a heterogeneous catalyst for biodiesel production. The characterization results demonstrated that MOF-801 possessed cubic structure, high crystallinity, good thermal stability, and moderate catalytic activity. Under optimal reaction conditions, the conversion rate of used vegetable oil was 60%; the activity of MOF-801 is probably due to the cationic Zr and anionic O_2_ sites in the crystal structure.

Besides this, de la Flor, et al. [32] reported the synthesis of defective UiO-66(Zr) catalyst for the production of jet-fuel precursors via aldol-condensation, and total furfural conversion and selectivity (~100%) were obtained. Rapeyko, et al. [33] also reported that the as-synthesized UiO-66 could efficiently catalyze the selective ketalization of levulinic acid and 1,2-propanediol, and high selectivity (91–93%) was attained.

From the studied literature, it is derived that the single Zr-based MOFs can be considered as a catalyst for acid-catalyzed reactions. However, the activity of single Zr-based MOFs still needs to be further improved, and facilely tuning the defect density on nodes by introducing modulators may be a very interesting approach to developing highly active Zr-based MOF catalysts.

### 2.2. Modified Zr-Based MOF Catalysts

To improve the catalytic activity and chemical stability of Zr-based MOFs, several researchers have investigated various types of modification methods, including functional organic linkers, loading active components (e.g., lipase, ionic liquids, heteropoly acid, etc.), and the incorporation of metal ions into Zr-based MOFs (Table 1).

**Table 1:** Recent findings on green fuel production using modified Zr-based MOF catalysts.

Entry	Raw Material	Catalyst	Reaction Conditions (Time, Temperature, Catalyst Amount, Molar Ratio (Acid(Oil):Alcohol))	Yield (*Y*/%) or Conversion (*C*/%)	Reusability	*E*a (KJ/mol)	Ref.
1	Lauric acid + Methanol	UiO-66-NH_2_	2 h, 60 °C, 8%, 1:26	Y > 99	Not reported	\	[34]
2	Levulinic acid + *n*-butanol	UiO-66-NH_2_	5 h, 120 °C, 1.8%, 1:6	Y = 99%	3 cycles, no significant loss	\	[35]
3	Levulinic acid + Ethanol	UiO-66-(COOH)_2_	24 h, 78 °C, 0.39%, 1:20	Y = 97%	5 cycles, Y = 93.9%	\	[36]
4	Oleic acid + Methanol	UiO-66(Zr)-NH_2_	4 h, 60 °C, 6%, 1:39	C = 97%	4 cycles, C > 50%	15.13	[37]
5	Oleic acid + Methanol	10SA/UiO-66(Zr)	4 h, 25 °C, 6%, 1:39	C = 94.5%	6 cycles, C = 83%	32.53	[38]
6	Levulinic acid + Ethanol	UiO66-SO_3_H(100)	6 h, 80 °C, 0.4%, 1:10	Y = 87%	4 cycles, Y = 84%	\	[39]
7	*Ricinus communis* oil + Methanol	Lipase/Zr-MOF/PVP	12 h, 50 °C, 2 mg, 1:3	C = 83%	7 cycles, C = 66%	\	[40]
8	Oleic acid + Methanol	UiO-G	2 h, 70 °C, 8%, 1:12	C = 91.3%	4 cycles, C = 66.6%	28.61	[41]
9	Acetic acid + Isooctyl alcohol	UiO-67-CF_3_SO_3_	18 h, 90 °C, 0.2 g, 6:1	C = 98.6%	5 cycles, C = 95.9%	\	[42]
10	Tripalmitin + Methanol	UiO-66-[C_3_NH_2_] [SO_3_CF_3_]	12 h, 85 °C, 0.025 g, 1:121.5	Y = 86.6–98.4%	Not reported	38.9	[43]
11	Jatropha oil + Methanol	PSH/UiO-66-NO_2_	4 h, 70 °C, 4%, 1:25	C= 97.57%	3 cycles, C= 77.14%	\	[44]
12	Oleic acid + Methanol	AIL@NH_2_-UiO-66	6 h, 75 °C, 5%, 1:14	C = 95.22%	6 cycles, C = 90.42%	\	[45]
13	Oleic acid + Methanol	Ca^2+^/UiO-66(Zr)	4 h, 60 °C, 6%, 1:39	Y = 98%	5 cycles, Y = 84%	36.73	[46]
14	Oleic acid + Methanol	K-PW_12_@UIO-66(Zr)	4 h, 75 °C, 5%, 1:20	C = 90%	10 cycles, no significant loss	\	[47]
15	Acetic acid + *n*-butanol	HPW@UiO-66	3 h, 120 °C, 3%, 1:2	C = 80.2%	4 cycles, C = 63%	\	[48]
16	Soybean oil + C8 + C10	Cs_2.5_H_0.5_PW_12_O_40_@UiO-66	10 h, 150 °C, 7%,1:5:5	FA incorporation =20.3%	5 cycles, no significant loss	\	[49]
17	Soybean oil + Methanol	AILs/HPW/UiO-66-2COOH	6 h, 110 °C, 10%, 1:35	C = 95.8%	5 cycles, C > 80%	\	[50]
18	Euphorbia Lathyris L. oil + Methanol	HPW/UiO-66-NH_2_	8 h, 180 °C, 3.5%, 1:40	Y = 91.2%	4 cycles, no significant loss	31.0	[51]
19	Oleic acid + Methanol	FDCA/SA-UiO-66(Zr)	24 h, 60 °C, 6%, 1:40	Y = 98.4%	6 cycles, Y > 90%	\	[52]
20	Soybean oil + Methanol	PW_12_@UIO-66	4 h, 75 °C, 0.2 g, 1 g:5.5 ml	C = 91.1%	4 cycles, no significant loss	\	[53]
21	Lauric acid + Methanol	HSiW-UiO-66	4 h, 160 °C, 7%, 1:20	C = 80.5%	4 cycles, C = 70.2%	27.5	[54]
22	Oleic acid + Methanol	ZrSiW/UiO-66	4 h, 150 °C, 8%, 1:20	C = 98.0%	4 cycles, C = 88.9%	\	[55]
23	Lauric acid + Methanol	Ag_1_(NH_4_)_2_PW_12_O_40_/UiO-66	3 h, 150 °C, 10%, 1:15	C = 75.6%	4 cycles, C = 70.6%	35.2	[56]
24	Oleic acid + Methanol	Ce-BDC@HSiW@UiO-66	4 h, 130 °C, 0.2 g, 1:30	C = 81.5%	6 cycles, C = 76.9%	\	[57]

**Figure 4:**
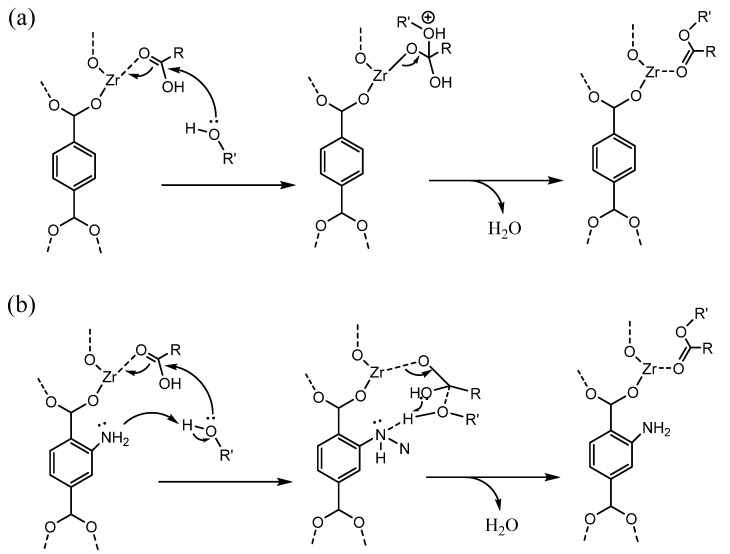
Plausible mechanisms for esterification: (**a**) UiO-66 catalyst; (**b**) UiO-66-NH_2_ acid–base catalyst. (Adapted with permission from Ref. [34]. Copyright 2015, Elsevier.)

Cirujano’s group [34,35] prepared an UiO-66-NH_2_ catalyst to convert lauric acid to methyl laurate via an esterification reaction. The high activity of UiO-66-NH_2_ with respect to UiO-66 is attributed to the occurrence of cooperative acid–base catalysis in the frame network. In addition, UiO-66-NH_2_ has been successfully used for esterification of levulinic acid with various alcohols. A possible bifunctional acid–base catalyst mechanism for esterification was proposed, as displayed in Figure 4.

Likewise, Wang, et al. [36] employed UiO-66-(COOH)_2_ as a heterogeneous catalyst for the esterification of levulinic acid, and Abou-Elyazed, et al. [37] also employed UiO-66(Zr)-NH_2_ for the esterification of oleic acid. Meanwhile, Abou-Elyazed’s group [38] also demonstrated the direct preparation of Ca^2+^-doped UiO-66(Zr) under solvent-free conditions. In detail, the introduction of Ca^2+^ could greatly enhance the catalytic performance and stability in the esterification because of the existence of double active sites with the formation of more defects.

Desidery, et al. [39] investigated partially and fully sulfonated hydrated UiO66 catalysts prepared by one-step solvothermal synthesis. Compared to that of commercial Amberlyst 15, the activity of the fully sulfonated hydrated UiO66 afforded the highest yield of ethyl levulinate.

UiO-66(Zr) used as a support for *p*-toluenesulfonic acid (PTSA) through a defect coordination strategy was proposed by Li, et al. [41]. Their results indicated that the PTSA was successfully introduced into UiO-66(Zr), and the highest conversion rate of oleic acid to biodiesel of 91.3% was acquired under mild conditions. More specifically, a reusability study showed that the conversion was dramatically reduced from 91.3% to 76.65% after four cycles, and they verified a loss of Zr and S in the reaction system.

**Figure 5:**
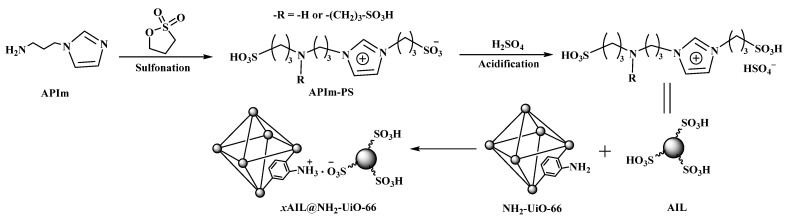
The suggested structures for AIL and *x*AIL@NH_2_-UiO-66 (where *x* indicates the mass ratio of AIL to NH_2_-UiO-66).

Recently, acidic or basic ionic liquids (ILs) have shown efficient catalytic activities in various organic reactions. However, they also suffer from several shortcomings, such as high viscosity, diffusion limitations, and difficulty in separation. In order to overcome these problems, the introduction of ILs into Zr-based MOFs has been studied [42,43,44,45]. Acidic ILs (AIL) were introduced into the NH_2_-UiO-66 matrix (See Figure 5) via acid–base interaction by Lu, et al. [45]. Accordingly, the best mass ratio of AIL to NH_2_-UiO-66 in 3AIL/NH_2_-UiO-66 displayed excellent activity and reusability in the esterification of oleic acid; a conversion rate of 95.22% was achieved in 6 h, and it could still reach 90.42% conversion after six cycles. Moreover, it was concluded that the good conversion rate was attributed to the stimulating synergy between the -SO_3_H group of AIL and the -NH_2_ group of NH_2_-UiO-66 on the MOFs.

Apart from active ILs, heteropoly acids (HPAs) with structural diversity and tunable Brønsted/Lewis acidity have also been reported as efficient acid catalysts for biodiesel synthesis. This is despite their high activity, solubility in many polar solvents, and very low surface area, which limit their application for catalysis. Therefore, the loading of various HPAs on Zr-based MOF materials has been performed [47,48].

Xie’s group [50] studied the one-pot transesterification–esterification of acidic vegetable oils to produce biodiesel by employing UiO-66-2COOH modified with HPW and sulfonated ILs as an acid catalyst (AILs/POM/UiO-66-2COOH). In their study, the surface area, pore volume, and mean pore size of the as-prepared composite catalyst were found to be 8.63 m^2^/g, 0.04 cm^3^/g, and 16.07 nm, respectively. Furthermore, the highest observed conversion rate was 95.8%, and the solid catalyst could maintain high activity even when 9 wt% free fatty acid and 3 wt% water were added into the feedstock.

As reported in much of the literature, Yang’s group [51] synthesized HPW/UiO-66-NH_2_ Lewis/Brønsted acid bifunctional hybrid catalyst by the electrovalent assembly of HPW and UiO-66-NH_2_. The resulting HPW/UiO-66-NH_2_ exhibited a highest acid density of 1.7 mmol/g, larger surface area of 301.6 m^2^/g, and both Lewis and Brønsted acid sites. The biodiesel yield obtained from the (trans)esterification of *Lathyris* L. oil was more than 91.2%. Notably, the composite catalyst was reusable for four cycles with no significant decrease in activity, and hot filtration experiments showed that the composite has heterogeneous characteristics. Another study conducted by Yang’s group [52] examined the synthesis of FDCA/SA-UiO-66(Zr) catalyst by a facile grinding method. Accordingly, DCA/SA-UiO-66(Zr) demonstrated superior or equivalent catalytic activity in the esterification of oleic acid due to its Lewis acidity and hydrophobicity.

Recently, our group also studied the production of biodiesel from free fatty acid with methanol over HPAs or doped HPAs incorporated into UiO-66 frameworks (e.g., ZrSiW/UiO-66, Ag_1_(NH_4_)_2_PW_12_O_40_/UiO-66, and Ce-BDC@HSiW@UiO-66) [54,55,56,57]. All these composite catalysts exhibited good catalytic activity and reusability. Table 1 summarizes the modified Zr-based MOF catalysts used for biofuel production. As can be seen here, many researchers agree that modified Zr-based MOF catalysts can effectively catalyze esterification or transesterification processes.

### 2.3. Zr-Based MOF-Derived Catalysts

Recently, MOFs have also been employed as a precursor substrate and template support for derived material synthesis. The synthesis of porous carbon and metal oxide via a thermal decomposition process was first reported by Xu’s group [58,59]. Since then, MOF derivatives have been attracted increasing attention as novel catalysts. In particular, the pyrolysis of defective Zr-based MOFs can provide a promising platform for various functional materials’ synthesis.

**Figure 6:**
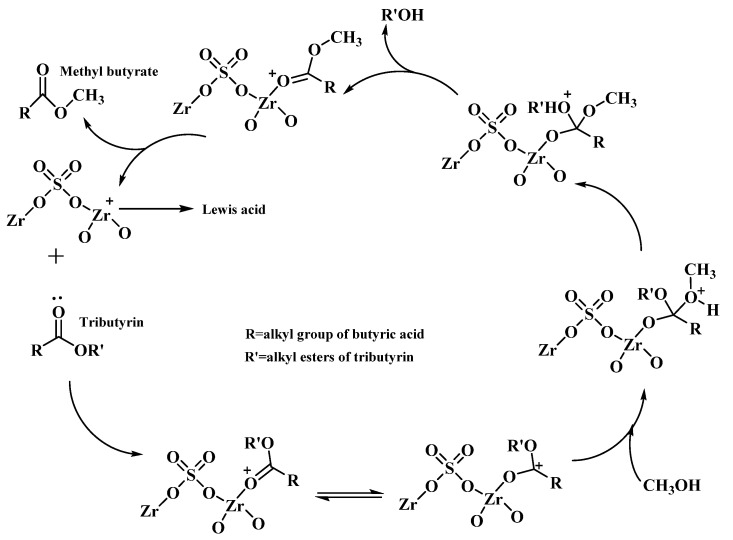
Reaction mechanism of transesterification on mesoporous sulfated zirconia nanosheets. (Adapted with permission from Ref. [60]. Copyright 2020, Royal Society of Chemistry.)

Lu, et al. [60] successfully synthesized flower-like mesoporous sulfated zirconia nanosheets via the thermal decomposition of in situ sulfated Zr-MOFs as the S/Zr ratio increased to 0.5. Investigations on the sulfated zirconia nanosheets at a calcination temperature of 500 °C showed a large surface area (186.1 m^2^/g) and strong interaction between the sulfate and zirconia atoms, affording excellent catalytic performance and stability for the production of biodiesel. Besides this, the mechanism of transesterification was studied, as shown in Figure 6.

Li, et al. [61] employed a UiO-66(Zr) support impregnated with calcium acetate for CaO/ZrO_2_ catalyst synthesis via an activation process in nitrogen (UCN) and air (UCA) atmosphere. Among these catalysts, UCN650 calcined at 650 °C attained a relatively large specific surface area (24.06 m^2^/g); meanwhile, the catalyst generated active sites of Ca*_x_*Zr*_y_*O*_x+2y_* and CaO inside and was shown to be effective in catalyzing palm oil transesterification, reaching a maximum conversion rate of 98.2%. Moreover, the UCN650 catalyst maintained its catalytic properties when it was recycled three times. The properties of the resulting biodiesel (density, kinetic viscosity, acid value, etc.) were also found to comply with the EN 14214 standards.

**Figure 7:**
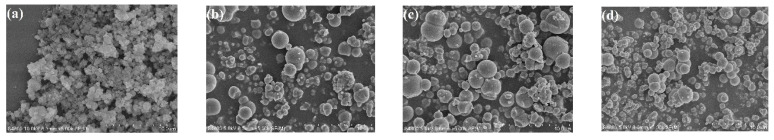
SEM images for (**a**) HSiW@UiO-66, (**b**) HSiW@ZrO_2_-300, (**c**) HSiW@ZrO_2_-400, and (**d**) HSiW@ZrO_2_-500. (Adapted with permission from Ref. [62]. Copyright 2021, open access from Royal Society of Chemistry.)

Our group also employed UiO-66 as a precursor for HSiW@ZrO_2_ hybrid synthesis, and SEM images of HSiW@UiO-66 at different calcination temperatures (300 °C, 400 °C, 500 °C) are shown in Figure 7. Nanoporous HSiW@ZrO_2_ was obtained by calcinating at 300 °C, exhibiting relatively high surface area (338 m^2^/g), appropriate pore size (2.5 nm), strong acidity (6.2 mmol/g), and the highest catalytic activity in the esterification of oleic acid; its conversion rate was high at 94.0% and stayed above 80% after nine catalytic cycles [62].

Dimethyl ether (DME) has gained attention for its application as a second-generation fuel, and it can be synthesized through a methanol dehydration process. Goda, et al. [63] used UiO-66 as a precursor for the synthesis of ZrOSO_4_@C catalyst. In the experiment, it was observed that ZrOSO_4_@C has weak and intermediate acidic sites and could be effectively applied for methanol dehydration to DME, with the highest conversion (100%) and selectivity (100%).

Hong, et al. [64] prepared 3D porous Cu@ZrO*_x_* catalysts via in situ reconstruction of size-confined Cu@UiO-66 for methanol synthesis from CO_2_ hydrogenation, and the optimized catalyst exhibited quite high methanol selectivity of 78.8% at 260 °C and 4.5 MPa, attributed to the many Cu^+^−ZrO*_x_* interfaces present as active sites in the material framework. Zeng’s group [65] also designed and prepared porous hydrous zirconia via UiO-66 pyrolysis as a support for Ni^II^ centers. In methane production from CO_2_ hydrogenation, the resultant catalyst exhibited excellent activity and stability.

Based on the literature, Zr-based MOF-derived materials with stable porous structures and many active sites are expected to be widely used for the development of high-performance composite catalysts in the future.

## 3. Conclusions and Future Prospects

Herein, a comprehensive review was presented on the synthesis of Zr-based MOFs and their derived composite materials for their catalytic application in green biofuel synthesis in recent years. The current review attempted to thoroughly demonstrate the use of single Zr-based MOF catalysts, modified Zr-based MOF catalysts, and Zr-based MOF-derived catalysts in the literature. Their high surface area, adjustable pore structure, acceptable recyclability, and strong acid sites obtained by tuning structural defects make Zr-based MOFs suitable for esterification or transesterification process.

However, looking ahead, many challenges for the large-scale application of Zr-based MOFs still exist, such as the design and development of inexpensive Zr-based MOFs at an industrial scale with high yields. The self-assembly mechanism of Zr-based MOFs and their derived materials is still unclear, and further exploration via both experimental and theoretical approaches is still required. The chemical and thermal stability of Zr-based MOFs is still not adequate and needs to be further improved. Facilely tuning the defect density on nodes by exploring new modification approaches could bring beneficial changes to the catalytic performance. By combining Zr-based MOFs with appropriate active materials such as enzymes, graphene derivatives, and magnetic substances, composite materials could be synthesized to improve their catalytic performance. Controlling the structure, composition, and distribution of the active component of Zr-based MOF-derived catalysts, while aiming to maintain the original structure of the Zr-based MOFs, still needs to be further studied.

As a whole, the application of Zr-based MOFs and catalysts derived from them is important not only for green biodiesel synthesis but also for the conversion of biomass. Despite facing many challenges, hopefully, the existing issues will be resolved sooner or later, and the application prospects of biorefineries will also be very bright.

## Data Availability

Not applicable.
